# Ophthalmic artery Doppler in the complementary diagnosis of preeclampsia: a systematic review and meta-analysis

**DOI:** 10.1186/s12884-023-05656-9

**Published:** 2023-05-12

**Authors:** Pollyanna F. M. Vaz de Melo, Leonardo Roever, Tânia M. S. Mendonça, Fabrício da Silva Costa, Daniel Lorber Rolnik, Angélica L. D. Diniz

**Affiliations:** 1 Department of Obstetrics and Gynaecology, Federal University of Uberlândia - UFU, Avenida Pará, 1720, Uberlândia, 38504-320 Minas Gerais Brasil; 2 Clinical Research, Federal University of Uberlândia-UFU, Uberlândia, Brazil; 3 Medical School of Federal, University of Uberlândia-UFU, Uberlândia, Brazil; 4 Maternal Fetal Medicine Unit, Gold Coast University Hospital and School of Medicine, Griffith University, Gold Coast, QLD Australia; 5 Department of Obstetrics and Gynaecology, Monash University, Melbourne, Australia

**Keywords:** Doppler, Ultrasound, Ophthalmic artery, Meta-analysis, Pregnancy, Preeclampsia

## Abstract

**Objective:**

To evaluate the accuracy of different parameters of the ophthalmic artery Doppler (OAD) in the complementary diagnosis of preeclampsia (PE).

**Methods:**

This meta-analysis adhered to the PRISMA guidelines. To investigate the mean difference in OAD values, peak systolic velocity (PSV), end-diastolic velocity (EDV), second systolic velocity peak (P2), resistance index (RI), pulsatility index (PI), and peak ratio (PR), between PE cases (overall and according to severity) and controls, random-effects meta-analyses were conducted for each Doppler parameter, with overall PE and mild and severe PE subgroups. Diagnostic performance and heterogeneity were evaluated with summary receiver operating characteristic (sROC) curves and 95% confidence intervals obtained with bivariate models.

**Results:**

Eight studies stratified the results into mild and severe or late and early PE, involving 1,425 pregnant women. PR and P2 had better diagnostic performance than the other indexes, with the PR of AUsROC at 0.885, the sensitivity of 84%, and specificity of 92%, with a low false-positive rate of 0.08 and the P2 with AUsROC of 0.926, the sensitivity of 85% and specificity of 88%. RI, PI, and EDV showed good performance and consistency across studies but lower AUsROC values of 0.833, 0.794, and 0.772, respectively.

**Conclusion:**

Ophthalmic artery Doppler is a complementary tool with good performance for the diagnosis of overall and severe preeclampsia, with high and best sensitivity and specificity when using PR and P2 parameters.

**Supplementary Information:**

The online version contains supplementary material available at 10.1186/s12884-023-05656-9.

## Background

Preeclampsia (PE) is a multisystemic and multifactorial syndrome with multiple phenotypes [1]. A systematic review of global data available between 2002 and 2010 showed that the incidence of PE ranged from 1.2 to 4.2% of all pregnancies and is responsible for high rates of maternal morbidity and mortality worldwide [2, 3]. It is important to highlight that highest rates of PE are described in countries with less socioeconomic development, which generates greater concern regarding available resources for diagnosing and managing the disease [2, 3].

At present, in spite of considerable research number regarding PE, there are several gaps in the pathophysiology, and a diversity of clinical forms and heterogeneity among populations, facts that make the diagnosis difficult [4, 5]. A significant progress in the accuracy of PE diagnosis, a particularly atypical form of the disease or the related complications, was described by serum measurement of the angio and antiangiogenic markers, including placental growth factor (PlGF) and soluble fms-like tyrosine kinase-1 (sFlt-1) [6]. Although they are important and validated markers in predicting the diagnosis and management of PE, these are not widely available in low-income countries, therefore it seems reasonable to expand the diagnostic tools, extending beyond the placenta to the analysis to the cardiovascular and cerebral compartments, especially in those patients who do not fill the current criteria for diagnosis.

The ophthalmic artery is an easily accessible vessel for Doppler assessment that provides information on the less accessible intracranial circulation [7]. Ophthalmic artery Doppler (OAD) is a tool that has been studied as an aid in the diagnosis of PE, since its pathogenesis is not limited to the placental bed but extends to cardiovascular and endothelial adaptations during pregnancy [8]. in addition, ultrasound is a diagnostic method already consolidated in clinical obstetric practice and widely available in low-income countries. Several studies have described changes in the velocity wave pattern of the ophthalmic artery in women with PE, compared with normotensive pregnant women, both prenatally and in the postpartum period, with signs of decreased impedance and orbital hyperperfusion, represented by high values of peak ratio (PR), the second peak of systolic velocity (P2) and decreased resistance index (RI) and pulsatility index (PI) [9–19]. However, there is no consensus in the literature about the best diagnostic parameter of OAD in pregnant women with PE. The aid of this ultrasound examination may be relevant if used in the hospital environment to diagnose patients with PE where this information is urgently needed to optimize care and facilitate early treatment, especially in severe cases. The present systematic review and meta-analysis aimed to investigate the accuracy of different parameters of ophthalmic artery Doppler in the diagnosis of PE.

## Methods

### Protocol and registration

This meta-analysis adhered to the Preferred Reporting Items for Systematic Reviews and Meta-Analysis (PRISMA) guidelines [20]. The protocol was prospectively registered with PROSPERO (International Prospective Register of Systematic Reviews), registration CRD42019134115.

### Information sources and search techniques

In the period between July 1995 and January 2022, we used a systematic search of articles regarding the use of OAD in women with suspected or confirmed diagnosis of PE published in MEDLINE/PubMed, EMBASE, Bireme, Lilacs, Scopus, Web of Science, Cochrane library, as well as gray literature (Google scholar, Medxriv and Open Grey), without restriction as to language or time. We used combinations of Medical Subject Headings terms in the databases, searching by title and abstract, without adopting methodological filters to avoid missing relevant articles [21]. The descriptors adopted in each database are listed in Supplement 1.

### Search strategy and study selection

The search strategy was broad, and studies related to the research question were exported to the Rayyan [22] platform, an application that assists in article selection. Two researchers (PVM and ALDD) independently and blindly screened the articles by titles and abstracts, also resolving duplicates. We adopted the following inclusion criteria: studies until January 2022 that included pregnant women aged 18–45 years, with singleton pregnancies without or with risk factors for PE, with a diagnosis of PE at the time of OAD, with or without a history of smoking, with OAD data in one or two eyes. Exclusion criteria were duplicate articles in the databases, case reports, reviews, editorials, and case series with a small number of cases, systematic reviews, meta-analyses, as well as studies of pregnant women with reported previous eye diseases, significant heart disease, or internal carotid artery disease.

### Data extraction

All data included in this review were derived from tables or main text, and the data were extracted from each study and checked by two authors independently (PVM and ALDD) to reduce the risk of error in data collection. For each study, the following variables were extracted: first author’s name, year of publication, title of manuscript, outcomes, total sample size, number of events, study type, and for each OAD parameter test, peak systolic velocity (PSV), end-diastolic velocity (EDV), second systolic velocity peak (P2), resistance index (RI), pulsatility index (PI) and peak ratio (PR), false positive, false negative, sensitivity, specificity, positive predictive value (PPV), negative predictive value (NPV) and area under the curve (AUROC), all with the respective 95% confidence intervals (CI). The reported threshold for diagnosis for the index test was also collected. The database of two of the studies (Freitas 2018 and Diniz 2008) was obtained by contacting the authors.

### Quality assessment of studies

Risk of bias and quality assessment were undertaken for all included studies based on Quality Assessment of Diagnostic Accuracy Studies-2 (QUADAS-2) [23], a diagnostic performance study assessment tool, by two authors independently (PVM, ALDD). QUADAS-2 evaluates studies within four key domains in risk of bias and applicability concerns: patient selection, index test, reference standard, and flow of patients through the study. Each study in the review was graded as having either a low, high, or unclear risk of bias for each domain.

### Statistical analysis

To investigate the mean differences in the various ophthalmic artery Doppler parameters between PE cases and pregnancies without complications during the prenatal period (normal group), we carried out meta-analyses using random-effects models with the DerSimonian and Laird method for estimation of the inverse variance.

Subgroup meta-analyses were conducted for mild/late-onset and severe/early-onset preeclampsia when subgroup estimates were available. Statistical heterogeneity was evaluated with the I2 statistic [24].

We then performed a univariate analysis of sensitivities and specificities, followed by assessment of the diagnostic performance and heterogeneity with summary receiver-operating characteristics (sROC) curves, obtained by fitting bivariate models to the data to estimate the summary sensitivity and specificity along with 95% confidence intervals [25]. In the absence of covariates, these models are equivalent to hierarchical (HSROC) models [26]. Due to the inclusion of fewer than ten studies in each separate analysis of different Doppler parameters and the diagnostic accuracy nature of the studies, assessment of publication bias was not possible. Statistical analyses were conducted with the mada [27] and metaphor [28] packages in the statistical environment R [29].

## Results

### Study selection and data extraction

**Fig. 1:**
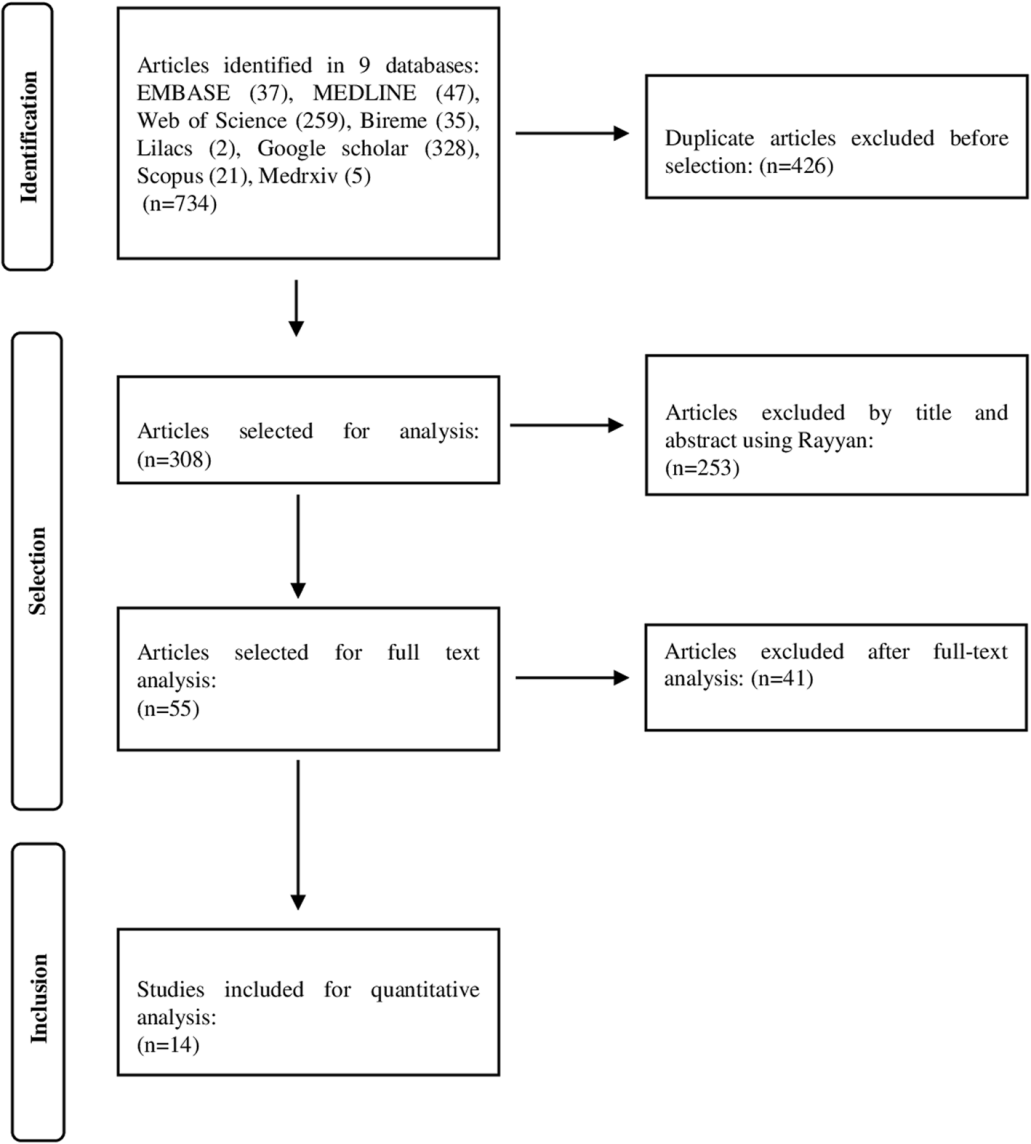
PRISMA Flowchart showing the identification, selection, eligibility and inclusion of primary studies

Of the 734 initially identified articles, 426 were duplicates, and 253 were excluded based on their titles and abstracts. Conflicts of selection were resolved by consensus of the two researchers, with a 16.7% rate of disagreement. There were 55 articles left for eligibility evaluation by full-text review, of which 41 were excluded by the following conditions: not meeting the inclusion criteria (*n* = 14), prediction studies (*n* = 10), and an outcome not related to the question (*n* = 17). Fourteen studies were included for qualitative and quantitative synthesis, totaling 1,425 patients (Fig. 1). Regarding the acquisition of the OAD parameters, there was divergence in the evaluation: five studies analyzed the average of the indexes between the two eyes, eight studies only one eye, and one did not provide information on this technical aspect.

### Study characteristics and risk of bias

**Fig. 2:**
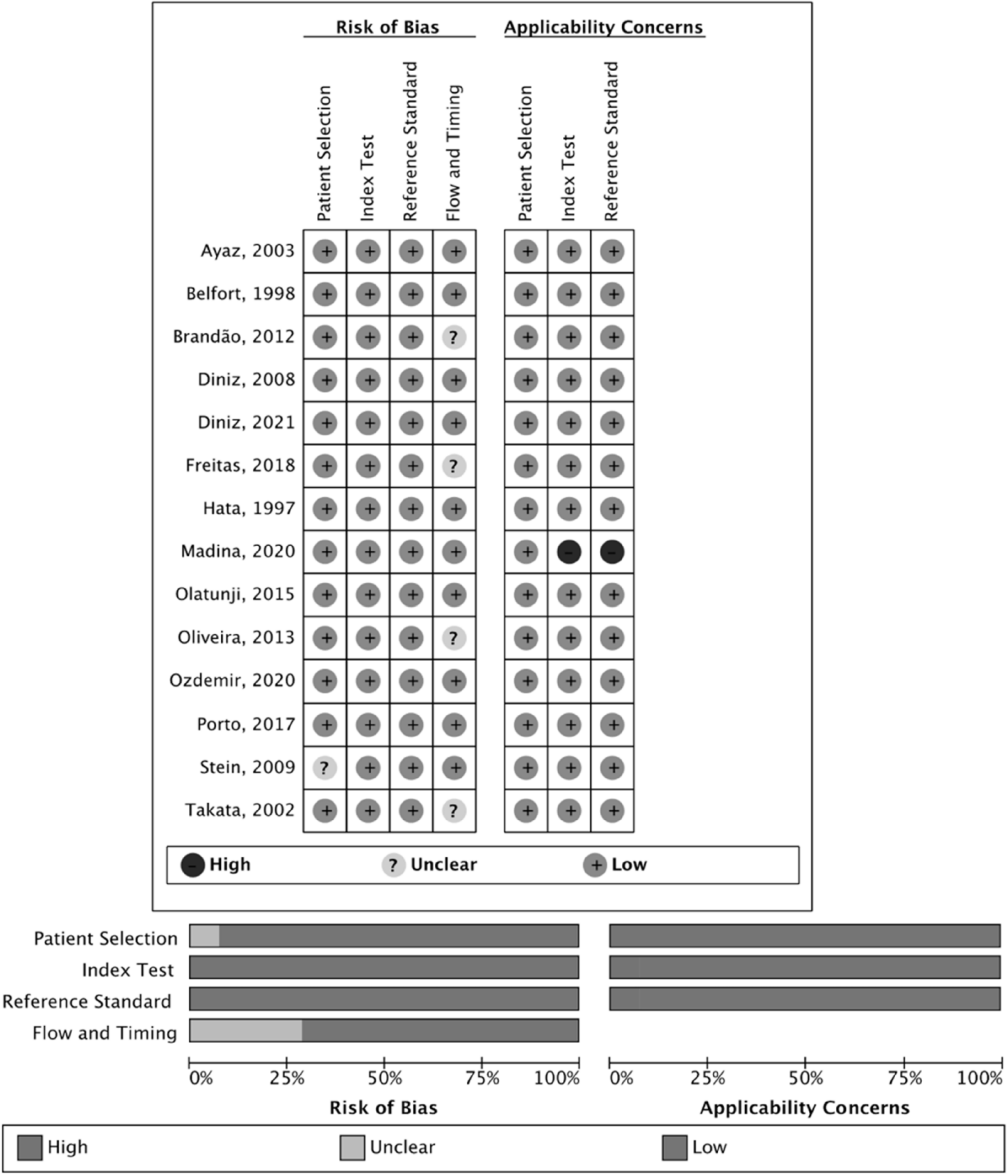
Use of the performance study assessment tool (QUADAS-2) to assess quality: summary of risk of bias and applicability. Only the first author of each study is given

The characteristics of the eligible studies are summarized in Table 1, and the risk of bias assessment is shown in Fig. 2. Five studies had uncertain risks in the patient selection and flow and timing domains [11, 30–33]. The study by Madina et al. [16] scored low and risk of bias in the assessment of applicability concerns involving the index and standard tests, as it did not specify how pregnant women were allocated or the parameters used to define preeclampsia in the methodology.

**Table 1:** Characteristics of the selected studies reporting the performance of ophthalmic artery Doppler in the diagnosis of preeclampsia

Studies	Study Design	Location	Population	Risk Factor	Diagnosis Criteria	Oda Parameters	n total	n PEa	n PEl	n PEg	n C
Hata 1997 [34]	Cross-sectional	Japan	IG:1-3 T	Not reported	^a^	PI	76	15	9	6	29
Belfort 1999 [17]	Cross-sectional	USA	IG: 3 T	Not reported	ACOG	RI	42	18	0	0	24
Takata 2002 [11]	Cross-sectional	Japan	IG: 3 T	No	NHBP2000	RI, PI, PVS, PR, EDV	99	52	25	27	32
Ayaz 2003 [19]	Cross-sectional	Turkey	IG: 3 T	No	^a^	RI, PI	60	30	30	0	30
Diniz 2008 [14]	Cross-sectional	Brazil	IG:25-35w	No	NHBP2000	RI, PI, PVS, PR, EDV	91	40	20	20	51
Stein 2009 [30]	Case control	Brazil	IG: 3 T	Yes	NHBP2000	RI, PI, PVS, PR, EDV, P2	32	16	0	0	16
Brandão2012 [31]	Cross-sectional	Brazil	IG: 2 T	No	NHBP2000	RI	81	56	30	26	25
Oliveira 2013 [32]	Cross-sectional	Brazil	IG:20-40w	No	NHBP2000	RI, PI, PR	349	60	30	30	289
Olantuji 2015 [15]	Prospective case control	Nigeria	IG: maior 20w	No	NHBP2000	RI, PI, PVS, PR, EDV, P2	92	42	24	18	50
Porto 2017 [18]	Cohort	Brazil	IG: 3 T	Yes	NHBP2000	RI	62	10		10	52
Freitas 2018 [33]	Cross-sectional	Brazil	IG: 3 T	No	ACOG	RI, PI, PVS, PR, EDV, P2, VM	65	36	23	13	29
Madina 2020 [16]	Cross-sectional	Pakistan	IG: 2 T e 3 T	Yes	Not reported	RI	60	30	0	0	30
Ozdemir 2020 [12]	Cross-sectional	Turkey	IG: após 20w	No	NHBP2000	RI, PI, PVS, PR, EDV, P2, S/D	100	50	0	0	50
Diniz 2022 [13]	Cohort	Brazil	IG: 20-41w	No	ISSHP2018	RI, PI, PVS, PR, EDV, P2	266	133	0	0	133

*GA* gestational age, *w* weeks, *1 T* first trimester, *2 T* second trimester; and *3 T* third trimester, *RI* resistance index, *PI* pulsatility index, *PVS* peak systolic velocity, *P2* second peak systolic velocity, *PR* peak ratio, *EDV* end-diastolic velocity, *PEa* all preeclampsia group, *PEl* mild preeclampsia group, *PEg* severe preeclampsia group, *C* control group, *UFU* Universidade Federal de Uberlândia, *UFMG* Universidade Federal de Minas Gerais, *UFRJ* Universidade Federal do Rio de Janeiro. Only the first author of each study is given

^a^Not reported, but they used the same criteria as preeclampsia defined as an elevated blood pressure of >  = 140/90 mmHg and proteinuria (2 +) on dipstick

### Meta-analysis

Of 14 studies, eight stratified PE into mild, severe, late, or early, grouped into mild/late PE or severe/early PE groups. Overall PE OAD parameters were those calculated in PE pregnant women not subdivided in mild/late, severe/late groups, defining the overall PE group. There were significant differences in the mean values of the PR, P2, RI, PI, and EDV parameters in overall, mild and severe PE cases between the studies. The summary of the mean difference, sensitivity/specificity, and sROC analyses of the OAD parameters for overall, mild and severe PE are shown in Table 2.

**Table 2:** Summary of the mean difference, sensitivity/specificity and sROC analyses of ophthalmic artery Doppler parameters for overall, mild and severe pre-eclampsia

Doppler	MD overall PE	MD mild PE	MD severe PE	Sensitivity	False-positive rate	AU sROC
**PR**	0.24 (0.14 a 0.35) *n* = 3 studies [12, 13, 30]	0.21 (0.11 a 0.30) *n* = 4 studies [11, 14, 32, 33]	0.35 (0.32 a 0.37) *n* = 4 studies [11, 14, 32, 33]	0.84 (0.77 a 0.89) *n* = 6 studies [13–15, 30, 32, 33]	0.08 (0.02 a 0.25) *n* = 6 studies [13–15, 30, 32, 33]	0.885 *n* = 6 studies [13–15, 30, 32, 33]
**P2**	9.67 (3.91 a 15.43) *n* = 4 [12, 13, 15, 30]	3.62 (0.93 a 6.31) *n* = 2 [15, 33]	4.26 (0. 39 a 8.14) *n* = 2 [15, 33]	0.85 (0.68 a 0.94) *n* = 4 [13, 14, 30, 33]	0.12 (0.06 a 0.23) *n* = 4 studies [13, 14, 30, 33]	0.926 *n* = 4 studies [13, 14, 30, 33]
**RI**	-0.07 (-0.10 a -0.03) *n* = 7 studies [12, 13, 16, 15, 17, 18, 20]	-0.02 (-0.14 a 0.09) *n* = 7 studies [11, 14, 15, 19, 31–33]	-0.12 (-0.14 a -0.09) *n* = 6 studies [11, 13, 15, 31–33]	0.76 (0.69 a 0.82) *n* = 7 studies [12–15, 30, 32, 33]	0.21 (0.15 a 0.28) *n* = 7 studies [12–15, 30, 32, 33]	0.833 *n* = 7 studies [12–15, 30, 32, 33]
**PI**	-0.48 (-0.59 a -0.38) *n* = 4 studies [12, 13, 15, 30]	-0.62 (-0.88 a -0.37) *n* = 7 [11, 14, 15, 19, 32–34]	-0.87 (-1.20 a -0.55) *n* = 6 studies [11, 14, 15, 32–34]	0.74 (0.66 a 0.80) *n* = 5 studies [13, 14, 30, 32, 33]	0.20 (0.11 a 0.33) *n* = 5 studies [13, 14, 30, 32, 33]	0.794 *n* = 5studies [13, 14, 30, 32, 33]
**PSV**	1.65 (-3.39 a 6.69) *n* = 4 studies [12, 13, 15, 30]	-0.36 (-3.32 a 2.60) *n* = 4 studies [11, 14, 15, 33]	1.51 (-3.67 a 6.69) *n* = 4 studies [11, 14, 15, 32]	0.55 (0.37 a 0.72) *n* = 3 studies [13, 14, 30]	0.46 (0.29 a 0.64) *n* = 3 studies [13, 14, 30]	0.556 *n* = 3 studies [13, 14, 30]
**EDV**	3.06 (0.79 a 5.34) *n* = 4 studies [12, 13, 15, 30]	2.37 (0.62 a 4.12) *n* = 4 studies [38, 40, 44, 46]	4.47 (1.27 a 7.66) *n* = 4 studies [11, 14, 15, 33]	0.77 (0.62 a 0.87) *n* = 4 studies [13, 14, 30, 3]	0.33 (0.22 a 0.46) *n* = 4 studies [13, 14, 30, 33]	0.772 *n* = 4 studies [13, 14, 30, 33]

*Abbreviations*: *MD* mean difference, *RI* resistance index, *PI* pulsatility index, *PVS* peak systolic velocity, *P2* second peak systolic velocity, *PR* peak ratio, *EDV* end-diastolic velocity Meta-analysis using random effects model for analysis of mean difference between ophthalmic artery Doppler indices, bivariate random effects model for sensitivity and specificity, and area under the summarized ROC curve

#### Peak Ratio (PR)

**Fig. 3:**
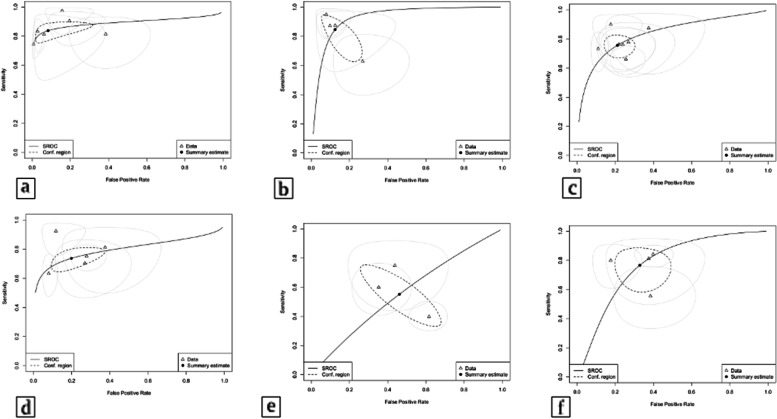
Summary sROC curve and estimated sensitivity (S) and false positive rate (FPR). **a** PR sROC: 0.885; S: 0.838 (95% CI 0.772;-0.888). FPR: 0.083 (95% CI 0.024;—0.251). **b** P2 sROC: 0.926, S: 0.846 (95% CI 0.680; 0.935), FPR: 0.124 (95% CI 0.063; 0.231); **c** sROC: 0.833, S: 0.757 (95% CI 0.688 – 0.815), FPR: 0.211 (95% CI 0.154 – 0.284); **d** sROC: 0.794, S: 0.737 (95% CI 0.664 – 0.799), FPR: 0.198 (95% CI 0.109 – 0.333, **e** sROC curve 0.556, S: 0.552 (95% CI 0.372 – 0.720), FPR: 0.461 (95% CI 0.293 – 0.639); **f** sROC curve 0.772, S: 0.767 (95% CI 0.623 – 0.868), PFR: 0.329 (95% CI 0.221 – 0.459)

In the overall PE group, there was a significant increase in PR in women without PE. This parameter was compared among cases and controls in three studies, and the mean difference was 0.24 (95% CI 0.15; 0.34). There was high heterogeneity, I2 = 90% (199 events), with cut-offs established by the primary studies of 0.75 [12], 0.81 [13], and 0.83 [30] (Supplement 2). In the mild PE group, there were four studies with a mean difference of 0.21 (95% CI 0.11; 0.30), I2 = 92% (98 events) with cut-offs of 0.65 [32], 0.70 [11], 0.71 [33], 0.81 [14], and in the severe PE group with four studies, the mean difference was 0.35 (95% CI 0.16; 0.38), I2 = 0% (90 events) with cut-offs of 0.81 [11], 0.84 [14], 0.85 [33], 0.89 [32] (Supplement 3). In six studies, sensitivity and specificity analysis showed that PR was one of the best performing indexes, with a sensitivity of 0.855 (95% CI 0.767; 0.913) and an E of 0.920 (95% CI 0.0778;0.974), with a low false-positive rate of 0.08 (Supplement 4). The summarized ROC curve for PR was 0.885 in six studies analyzed (Fig. 3).

#### Second systolic velocity peak (P2)

In the overall PE group, there was a significant increase in P2 in relation to women without PE. In four studies, the analysis of the overall PE group demonstrated that the mean difference was 9.68, CI (95% CI 4.92; 14.4), I2 = 89% (241 events), with cut-offs established by the primary studies of 23.99 cm/s [15], 30.44 cm/s [13], 33.90 cm/s [30], and 35.00 cm/s [12] (Supplement 5). In the mild PE group with two studies, the mean difference was 3.62 (95% CI 0.93; 6.31), I2 = 0% (47 events), with cut-offs established by the primary studies of 22.30 cm/s [33] and 22.62 cm/s [15] (Supplement 6). In the severe PE group, analyzing two studies, the mean difference was 4.26 (95% CI 0.39; 8.14), I2 = 0% (31 events), with cut-offs of 8.09 cm/s [15] and 14.20 cm/s [33] (Supplement 6). In four studies, P2 had a sensitivity of 0.857 (95% CI 0.718; 0.934), specificity of 0.880 (95% CI 0.718; 0.927) (Supplement 7) and AUC of 0.926 (Fig. 3).

#### Resistance index (RI) and pulsatility index (PI)

The PI and RI parameters showed good performance and consistency between studies but had lower accuracy than PR and P2. The PI and RI were lower in the PE group.

In seven studies evaluated, the mean RI difference for overall PE was -0.07 (95% CI -0.09; -0.04), I2 = 81% with cut-offs established by the primary studies of 0.62 [15], 0.64 [16], 0.66 [12, 17], 0.67 [18], and 0.68 [13, 30] (Supplement 8). In the mild PE group, there were seven studies with a mean difference of -0.02 (95% CI -0.14; 0.09), I2 = 99% with cut-offs of 0.63 [31], 0.641 [4], 0.66 [15], 0.73 [32, 33], 0.77 [11], and 0.97 [19] (Supplement 9). In a severe PE group with seven studies, the mean difference was -0.12 (95% CI -0.14; -0.09), I2 = 61% with cut-offs of 0.59 [15], 0.63 [32, 33], 0.64 [14], 0.65 [31], and 0.70 [11] (Supplement 9). In seven studies, the sensitivity was 0.765 (95% CI 0.692; 0.826) (Supplement 10), the pooled specificity was 0.793 (95% CI 0.724; 0.848), and the AUC was 0,833 (Fig. 3).

In four studies evaluated, the mean PI difference between overall PE and controls was -0.48 (95% CI -0.59; -0.37), I2 36% with cut-offs established by the primary studies of 1.17 [15], 1.28 [12], 1.29 [30], and 1.30 [13] (Supplement 11). In the mild PE group, there were seven studies with a mean difference of -0.62 (95% CI -0.88; -0.37), I2 = 95% with cut-offs of 0.91 [19], 1.24 [14], 1.47 [15], 1.61 [34], 1.62 [33], 1.63 [32], and 1.66 [11] (Supplement 12). In a severe PE group with six studies, the mean difference was -0.87 (95% CI -1.20; -0.55), I2 = 94% with cut-offs of 1.00 [14], 1.02 [15], 1.13 [34], 1.14 [33], 1.17 [34], and 1.61 [11] (Supplement 12). In five studies, the pooled sensitivity was 0.768 (95% CI 0.658; 0.851), specificity was 0.813 (95% CI 0.692; 0.894) (Supplement 13) and AUC was 0.794 (Fig. 3).

#### Peak systolic velocity (PSV) and end-diastolic velocity (EDV)

The PSV and EDV parameters showed variable results. PVS showed low accuracy, and EDV demonstrated higher velocities in the PE group and good accuracy. The mean PSV difference for overall PE (four studies) was 1.71 (95% CI -2.88; 6.29), I2 = 76% with cut-offs of 28.96 [15], 37.60 [13], 40.35 [30], and 47.00 [12] cm/s. In the mild PE group (four studies), the mean difference was -0.36 (95% CI -3.32; 2.60), I2 = 48% with cut-offs of 28.44 [15], 30.60 [32], 34.35 [14], and 48.30 [11] cm/s. In women with severe PE (four studies), the mean difference was 1.51 (95% CI -3.67; 6.69), I2 = 79% with cut-offs of 27.801 [5], 30.00 [32], 41.021 [4], 48.30 [11] cm/s (Supplement 14). Among the three studies, the pooled sensitivity was 0.551 (95% CI 0.376; 0.715), the specificity was 0.515 (95% CI 0.366; 0.662) (Supplement 15) and the AUC was 0.556 (Fig. 3).

The mean EDV difference for overall PE (four studies) was 3.09 (95% CI -1.13; 5.05), I2 = 84% with cut-offs of 9.90 [30], 7,45 [13], 7.90 [15], 9.20 [12] cm/s. In the mild PE group (four studies), the mean difference was 2.37 (95% CI 0.62; 4.12), I2 = 76% with cut-offs of 8.20 [33], 9.51 [15], 11.92 [14], and 12.90 [11] cm/s. In the severe PE group (four studies), the mean difference was 4.47 (95% CI 1.27; 7.66), I2 = 88% with cut-offs of 10.31 [15], 10.80 [33], 13.70 [11], and 16.07 [14] cm/s (Supplement 16). In four studies, the pooled sensitivity was 0.773 (95% CI 0.653; 0.861), the specificity was 0.675 (95% CI 0.560; 0.771) (Supplement 17), and the AUC was 0.772 (Fig. 3).

## Discussion

### Main findings

The analysis of DAO performance for the identification of PE cases was the focus of the current study. Five DAO parameters (PR, P2, PI, RI, EDV) demonstrate good diagnostic accuracy; however, PR and P2 stood out with higher sensitivity (0.855 and 0.857, respectively) and specificity (0.922 and 0.880, respectively). Therefore, PR and P2 are the best parameters and can be used as complementary tools in the diagnosis of PE, as well as in the detection of severe forms of PE, with more severe disease associated with larger differences between affected women and controls.

To our knowledge, this is the first systematic review with a meta-analysis conducted to test the diagnostic performance of OAD, including a total of 1,425 patients. The study involved pregnant women who were mainly in their late second and early third trimesters. The gestational age diversity does not become problematic, since the literature confirms that there are no changes in OAD indices as gestation advances [35, 36].

Regarding OAD as a diagnostic test, previous studies described that it has high intraobserver repeatability and interobserver reproducibility [37, 38]. Most studies described similar techniques used to perform the OAD, except for the study of Hata et al. [34], in which the OAD was evaluated with the pregnant women positioned in left lateral decubitus.

Concerning the studies included in this meta-analysis, it is noteworthy that there was representation from four continents, with seven Brazilian studies, two conducted in Turkey, one in Nigeria, one in the United States, two in Japan, and one in Pakistan, including diverse populations, minimizing the risk of a possible population bias and increasing the generalizability of the findings. The population of most of the primary studies had no risk factors for preeclampsia, as patients without previous endothelial damage and who did not use prophylaxis for the disease in the first trimester were included.

One of the strengths of this study was the broad search strategy that explored several databases, including data from the gray literature, in an attempt to minimize publication bias. In addition, the number of preeclampsia cases included in the analysis of the parameters was enough to address the question about the accuracy of the method. We also emphasize the low heterogeneity detected in the subgroup analysis of the PR and P2 parameters (I2 = 0%) in the identification of severe forms of PE, as well as the narrow confidence interval of the PR to severe forms of PE, which reduces the chance of downgrading the evidence detected even though we are facing meta-analyses of mostly cross-sectional studies. These findings of the severe PE form open an interesting window of opportunity regarding the implementation of multivariate models such as FULL-PIERS (Pre-eclampsia Integrated Estimate of Risk), which employ several maternal clinical and laboratory data to identify the risk of maternal adverse outcomes in cases of severe PE [39]. It is important to be aware that in the FULL-PIERS model, there is no parameter that addresses central nervous system changes as a diagnostic strategy for impending eclampsia.

### Limitations

The criteria adopted by the authors of the articles included defining PE in the population studied as a variable, which probably influenced the heterogeneity of the results and the comparison between the means of the parameters. Most manuscripts included in this meta-analyses adopted the PE classic criteria: blood pressure ≥ 140/90 mmHg with proteinuria > 300 mg in a 24-h urine sample (or 1 + on disptick random urine test or protein-to-creatinine ratio of 0.30 mg/mg) after the 20^th^ week of gestation. However, there are some modifications in the parameters used to define PE over the years. The same heterogeneity was not evident when comparing mild and severe forms of PE to uncomplicated pregnancies. The criteria used to define the mild and severe PE forms were similar and probably this influenced positively with low inconsistency rates when analyzing the comparison of the PR and P2 between the groups of severe PE and healthy women. Considering the acquisition of Doppler parameters, there was a divergence of the OAD evaluation; we identified five studies that analyzed the mean of the indexes between the two eyes of pregnant women, eight studies of only one eye, and one study that did not report these data. We believe that future studies with longitudinal designs and with Doppler evaluation of both eyes and homogeneous disease definitions are necessary to better estimate the role of OAD in the prediction and diagnosis of PE, particularly in low-resource settings where this relatively simple procedure may be applicable as a point of care test. The high heterogeneity observed for all AOD parameters in overall PE group can be explained because studies included different proportions of mild and severe cases and had smaller confidence intervals and greater statistical power.

### Interpretation and clinical implications

The diagnosis of PE has been undergoing adaptations over the years, based on a better understanding of its complexity and a broad spectrum of clinical presentation, all to improve the old simplistic definitions of the disease [1, 4, 40, 41]. The centralization of its pathogenesis in the placental region has been contrasted by the concept that cardiovascular and endothelial dysfunctions also participate and contribute to the development of hypertensive disorders during pregnancy [42]. In addition, there are cases in which diagnosis is difficult because the signs and symptoms do not fit the classical concepts described so far [43]. Considering that it seems reasonable to expand the range of diagnostic tools of the disease, with extension to the analysis of the cardiovascular and cerebral compartments, especially in those patients who do not meet the diagnostic criteria currently established. In addition, any evaluation of new diagnostic tests should be guided by how well these tools predict adverse outcomes rather than correlate with other definitions of the disease. The OAD may become a tool with good accuracy, adequate reproducibility, and low cost in low-income countries.

One potential practical applicability would be in the emergency room when the management of pregnant women with acute hypertensive disorders or cases of preeclampsia like syndromes, and the immediate differential diagnosis must be decisive for the first care. In this context, the authors also highlight that would already extend the scan to perform OA Doppler to identify the maternal cerebral vascular pattern since the AO Doppler is a fast, safe, reproducible, and easy- to-access method. This sonographic technique could also be used to determine the cerebrovascular effects of anticonvulsant and antihypertensive therapies in PE [44]. OA Doppler will never replace the current diagnostic criteria, but advancing with new studies on the hemodynamic pattern of OA may help in the diagnostic management in specific cases, where classic patterns are not identified [13].

The PR and P2 parameters stood out as those with higher accuracy in the diagnosis of PE. These two parameters analyze the systolic velocities of the AO, which highlight the hump-shaped morphology of the flow velocity wave. There are pathophysiological models currently described in the literature to justify these changes in the systolic phase of this peripherally accessed central nervous system artery [45].

## Conclusion

Ophthalmic artery Doppler is a complementary tool with good performance for the diagnosis of overall and severe preeclampsia, with the high and best sensitivity and specificity when using PR and P2 parameters.

## Supplementary Information

**Additional file 1.** 

**Additional file 2.** 

**Additional file 3.** 

**Additional file 4.** 

**Additional file 5.** 

**Additional file 6.** 

**Additional file 7.** 

**Additional file 8.** 

**Additional file 9.** 

**Additional file 10.** 

**Additional file 11.** 

**Additional file 12.** 

**Additional file 13.** 

**Additional file 14.** 

**Additional file 15.** 

**Additional file 16.** 

**Additional file 17.** 

## Data Availability

All data and materials described in the manuscript, including all relevant raw data, will be freely available to any scientist wishing to use them for non-commercial purposes, without breaching participant confidentiality. Angélica Lemos Debs Diniz must be contacted if someone wants to request the data from this study.
