# Supplementary figures and images for: Ophthalmic artery Doppler in the complementary diagnosis of preeclampsia: a systematic review and meta-analysis
Source: BMC Pregnancy Childbirth. 2023 May 12;23:343. doi: 10.1186/s12884-023-05656-9 (PMC10176747; doi:10.1186/s12884-023-05656-9)

## Slide 1
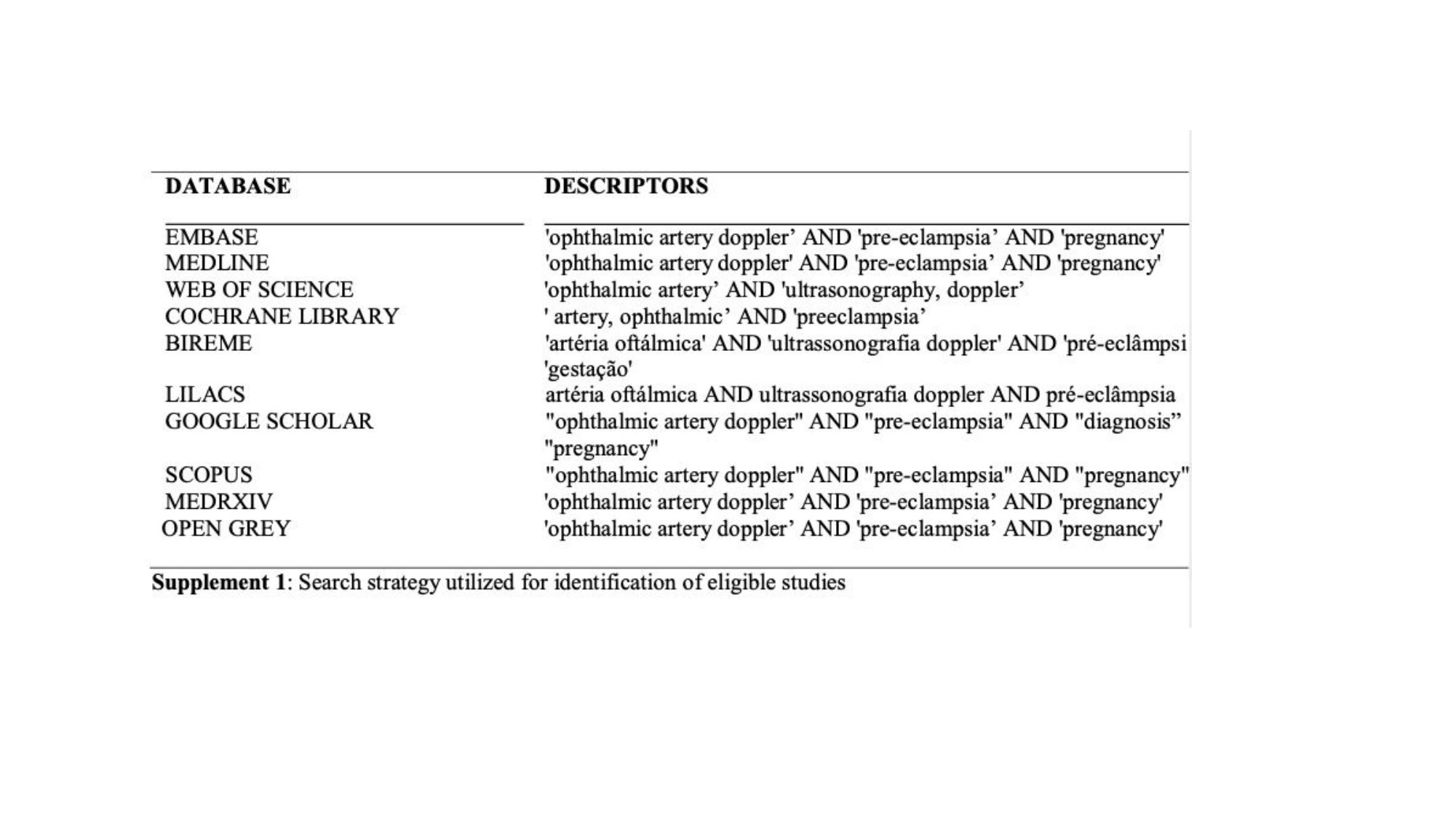

Supplement: Supplementary file 1 — Additional file 1. [file 12884_2023_5656_MOESM1_ESM.pptx]

## Slide 1
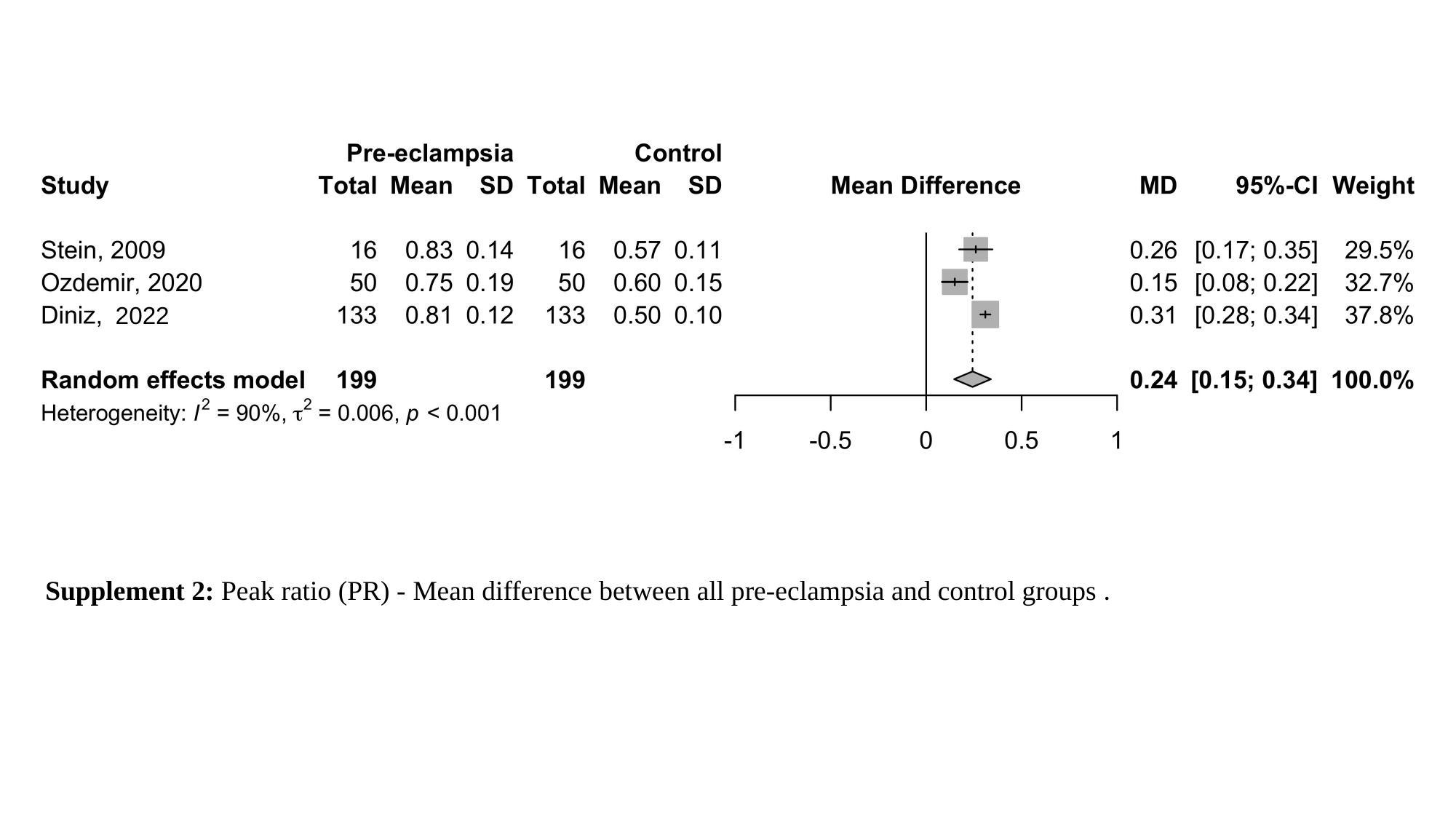

2022
Supplement 2: Peak ratio (PR) - Mean difference between all pre-eclampsia and control groups .

Supplement: Supplementary file 2 — Additional file 2. [file 12884_2023_5656_MOESM2_ESM.pptx]

## Slide 1
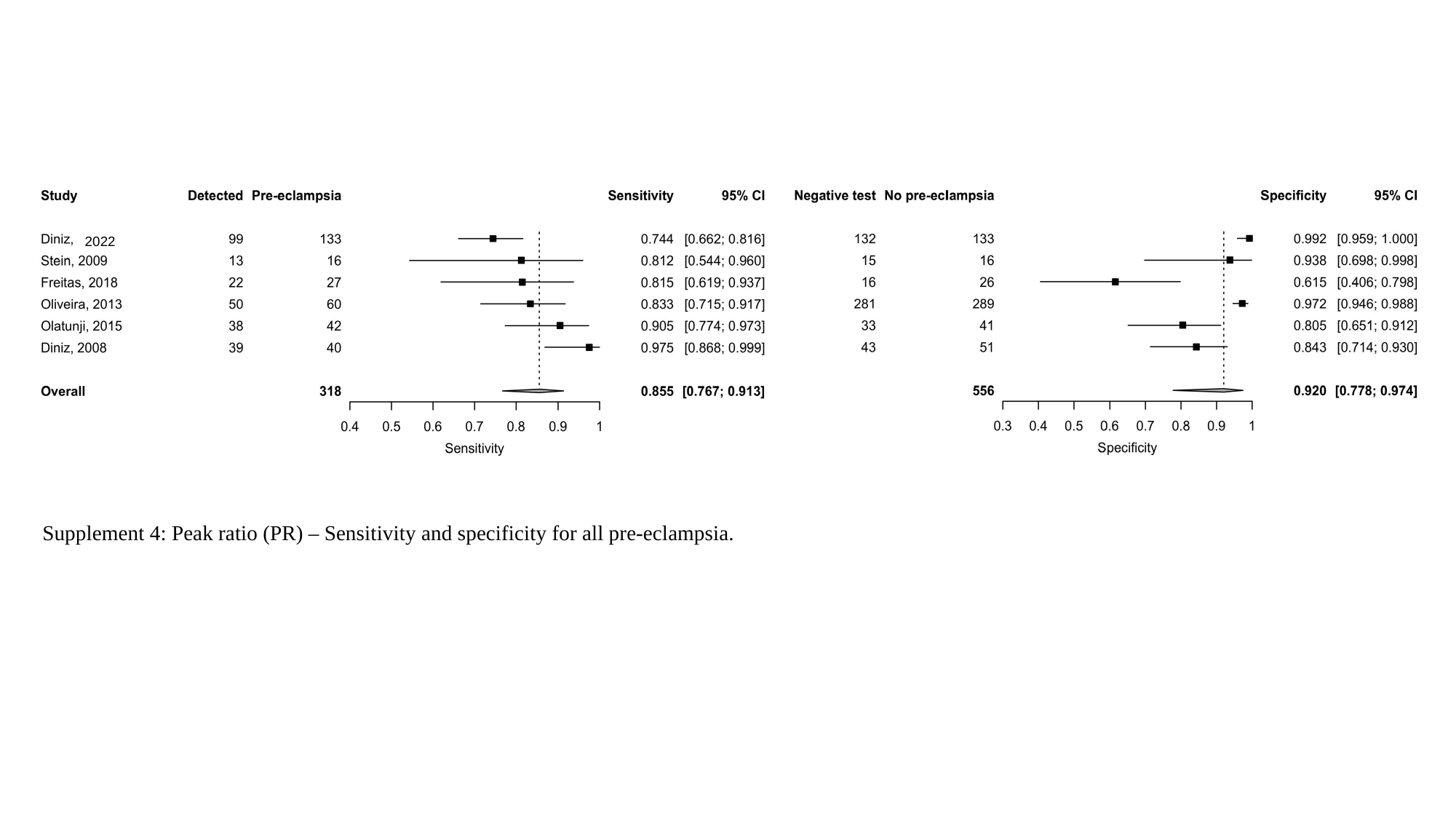

2022
Supplement 4: Peak ratio (PR) – Sensitivity and specificity for all pre-eclampsia.

Supplement: Supplementary file 4 — Additional file 4. [file 12884_2023_5656_MOESM4_ESM.pptx]

## Slide 1
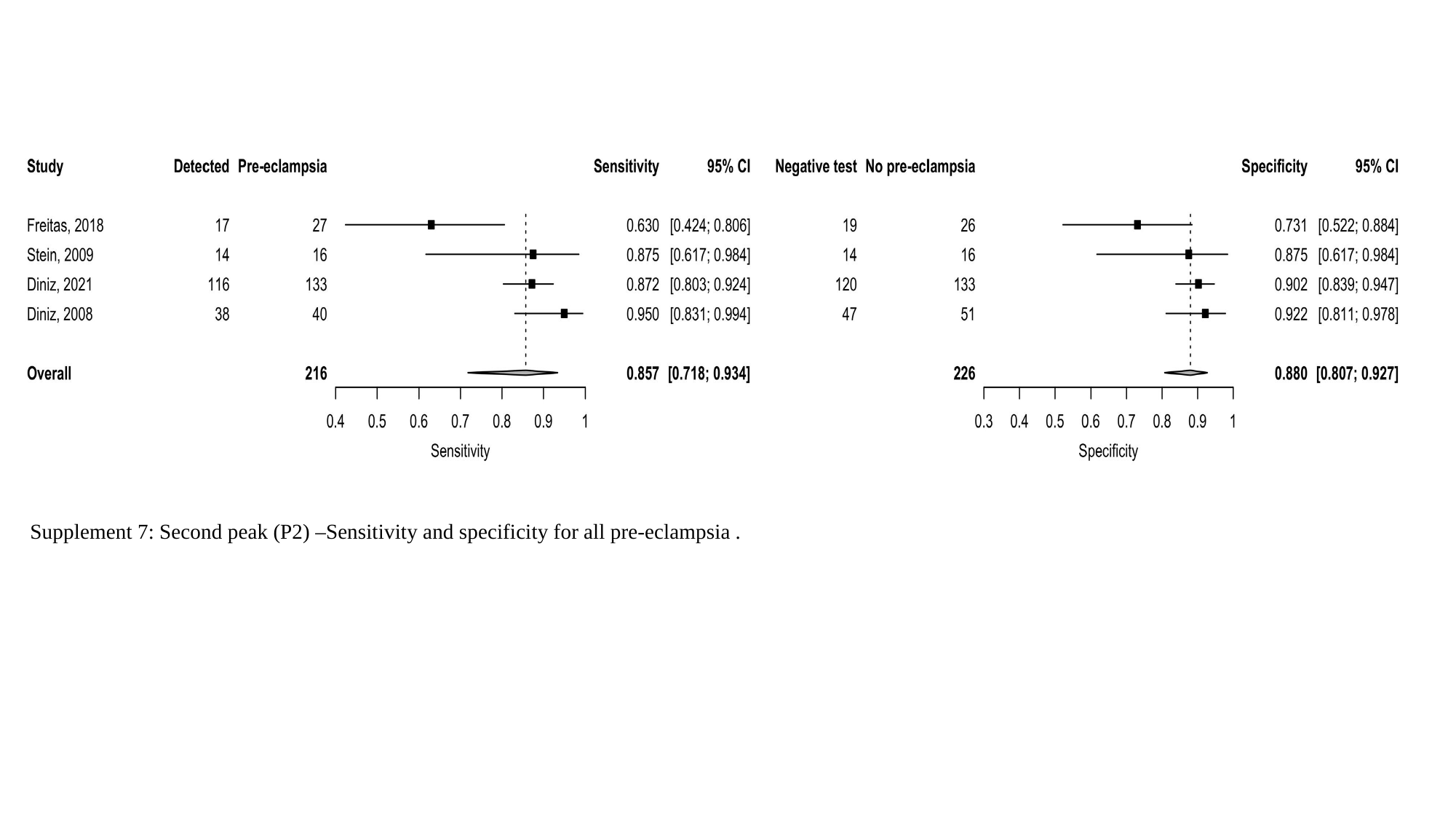

Supplement 7: Second peak (P2) –Sensitivity and specificity for all pre-eclampsia .

Supplement: Supplementary file 7 — Additional file 7. [file 12884_2023_5656_MOESM7_ESM.pptx]

## Slide 1
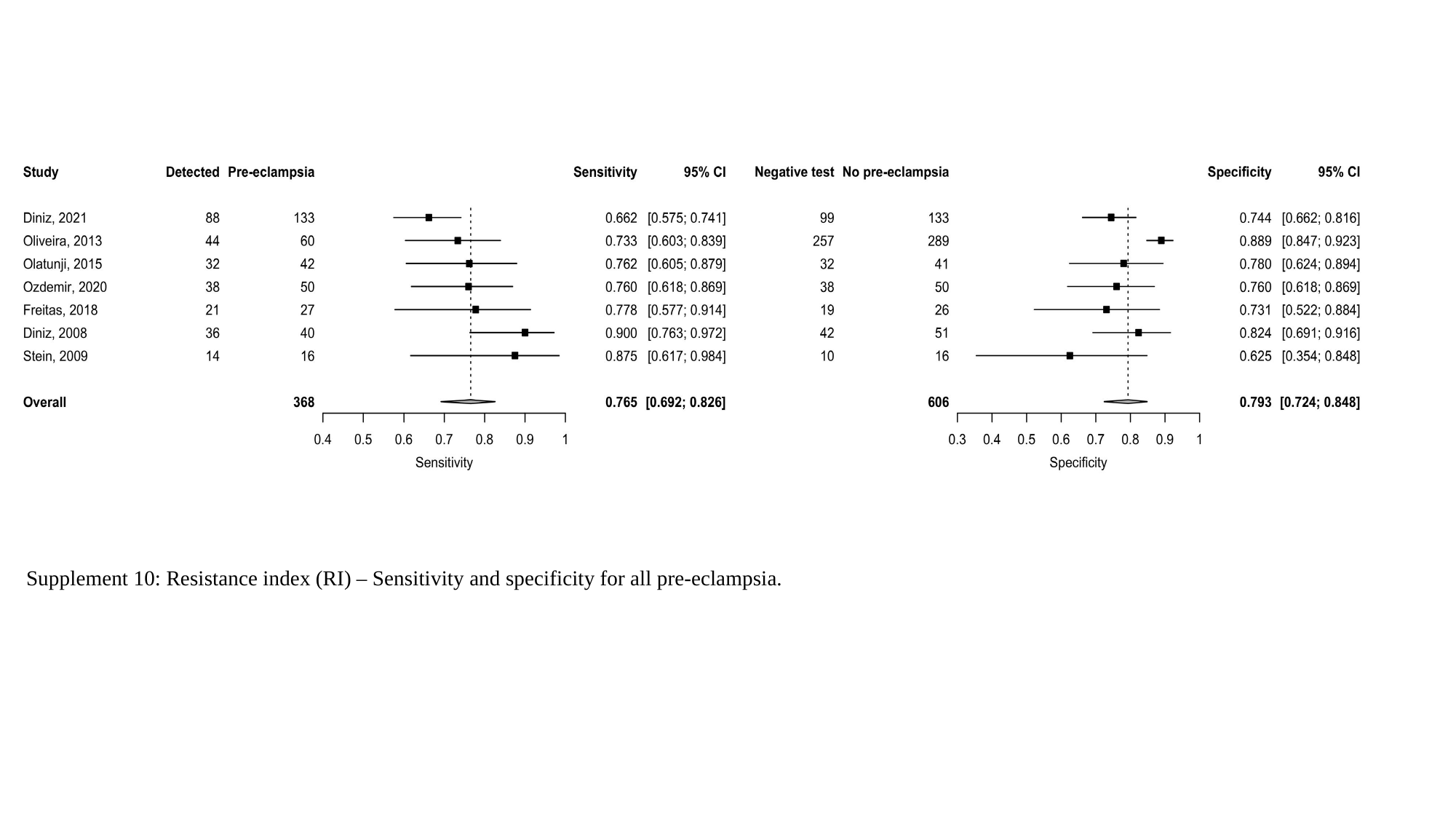

Supplement 10: Resistance index (RI) – Sensitivity and specificity for all pre-eclampsia.

Supplement: Supplementary file 10 — Additional file 10. [file 12884_2023_5656_MOESM10_ESM.pptx]

## Slide 1
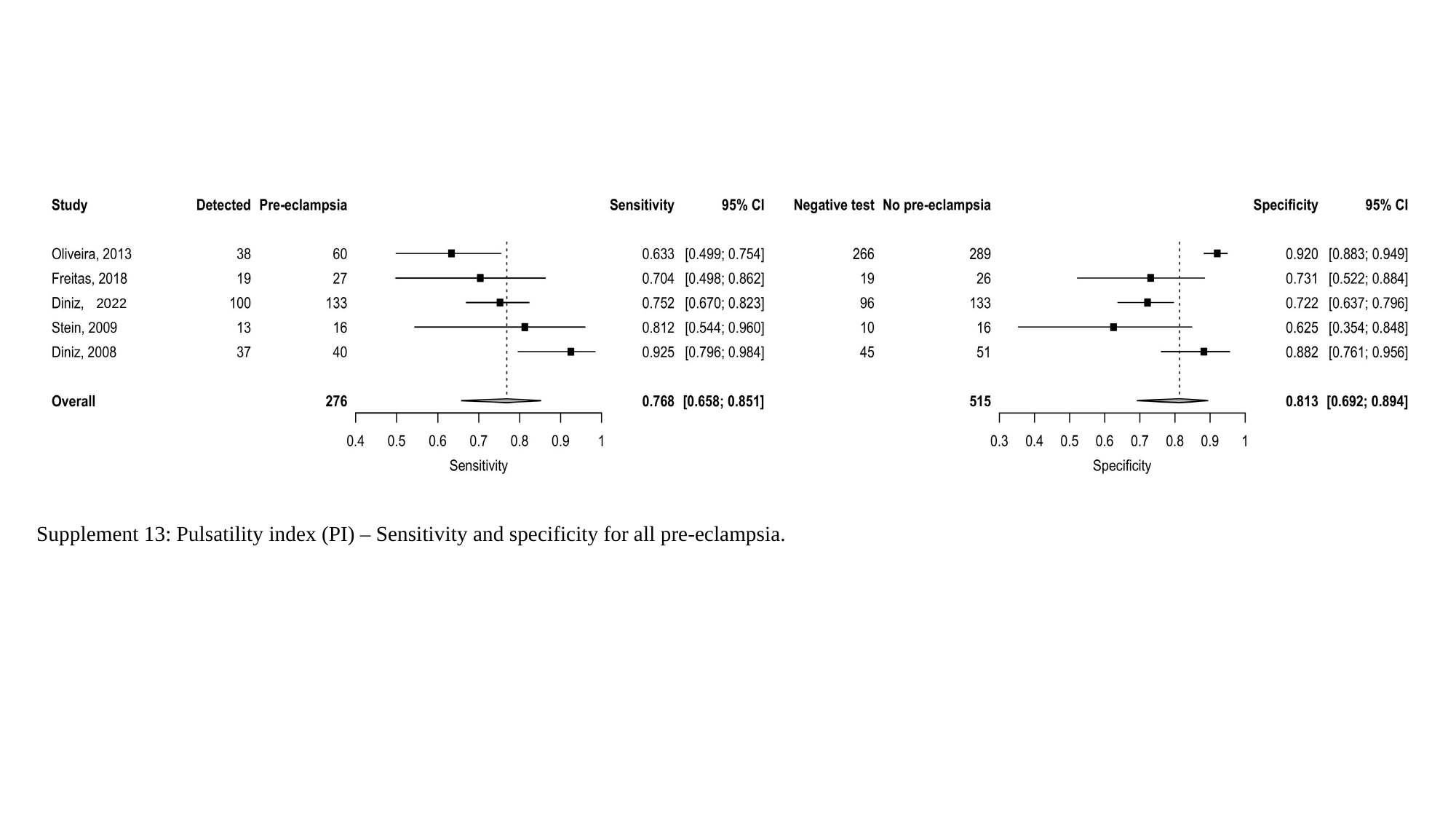

2022
Supplement 13: Pulsatility index (PI) – Sensitivity and specificity for all pre-eclampsia.

Supplement: Supplementary file 13 — Additional file 13. [file 12884_2023_5656_MOESM13_ESM.pptx]

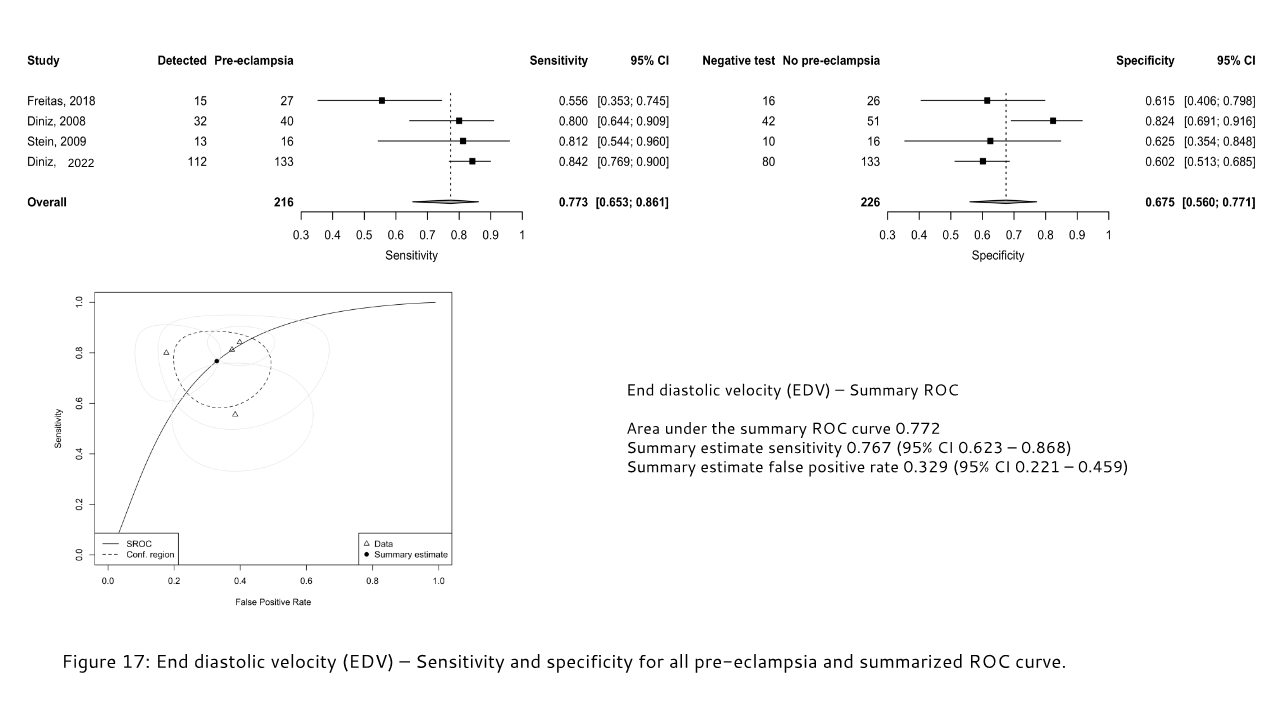

Supplement: Supplementary file 17 — Additional file 17. [file 12884_2023_5656_MOESM17_ESM.tiff]
