# Supplementary material for: Ophthalmic artery Doppler in the complementary diagnosis of preeclampsia: a systematic review and meta-analysis
Source: BMC Pregnancy Childbirth. 2023 May 12;23:343. doi: 10.1186/s12884-023-05656-9 (PMC10176747; doi:10.1186/s12884-023-05656-9)
Supplement: Supplementary file 5 — Additional file 5. [file 12884_2023_5656_MOESM5_ESM.pptx]

## Slide 1
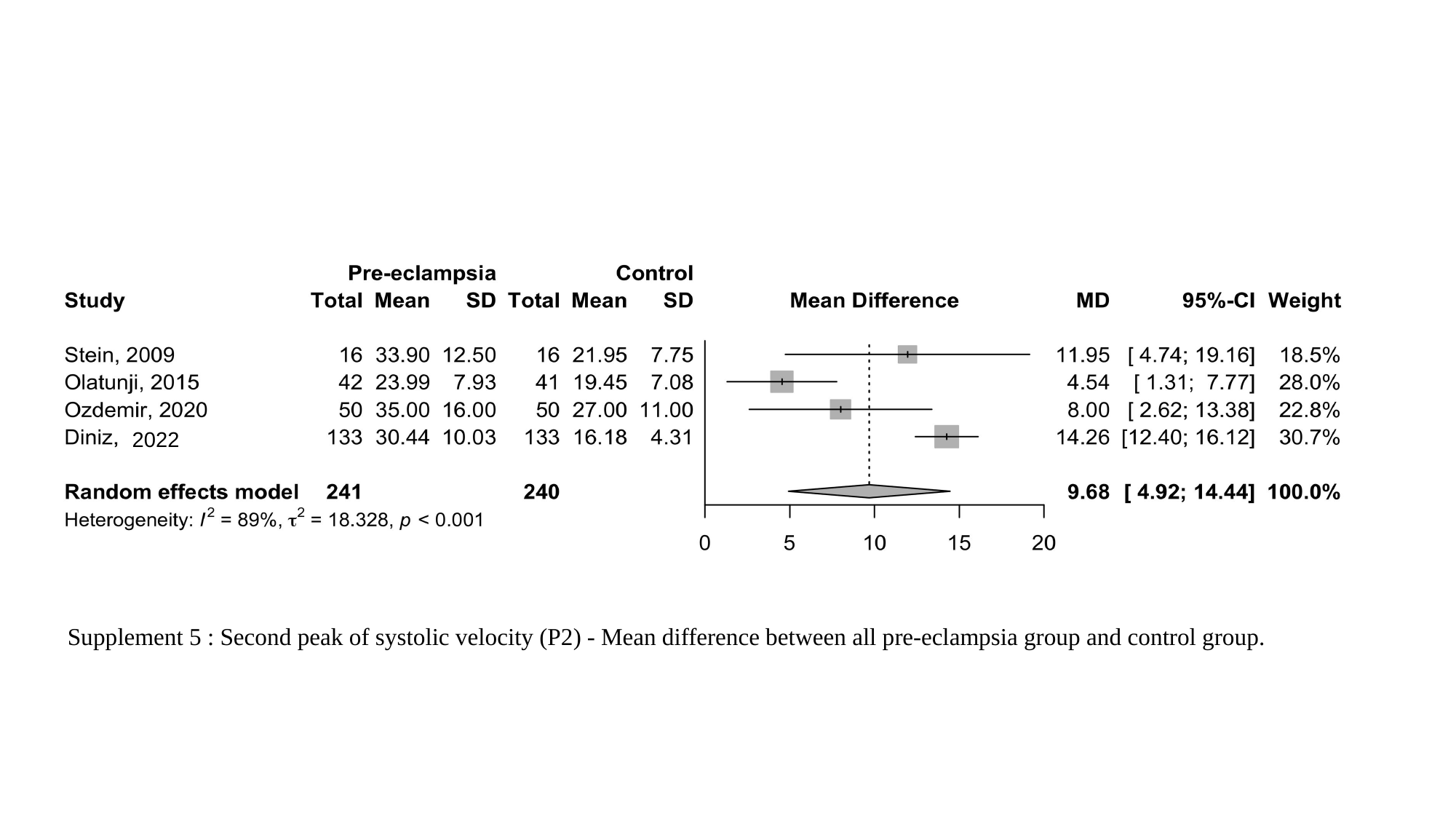

2022
Supplement 5 : Second peak of systolic velocity (P2) - Mean difference between all pre-eclampsia group and control group.
