# Supplementary material for: Ophthalmic artery Doppler in the complementary diagnosis of preeclampsia: a systematic review and meta-analysis
Source: BMC Pregnancy Childbirth. 2023 May 12;23:343. doi: 10.1186/s12884-023-05656-9 (PMC10176747; doi:10.1186/s12884-023-05656-9)
Supplement: Supplementary file 8 — Additional file 8. [file 12884_2023_5656_MOESM8_ESM.pptx]

## Slide 1
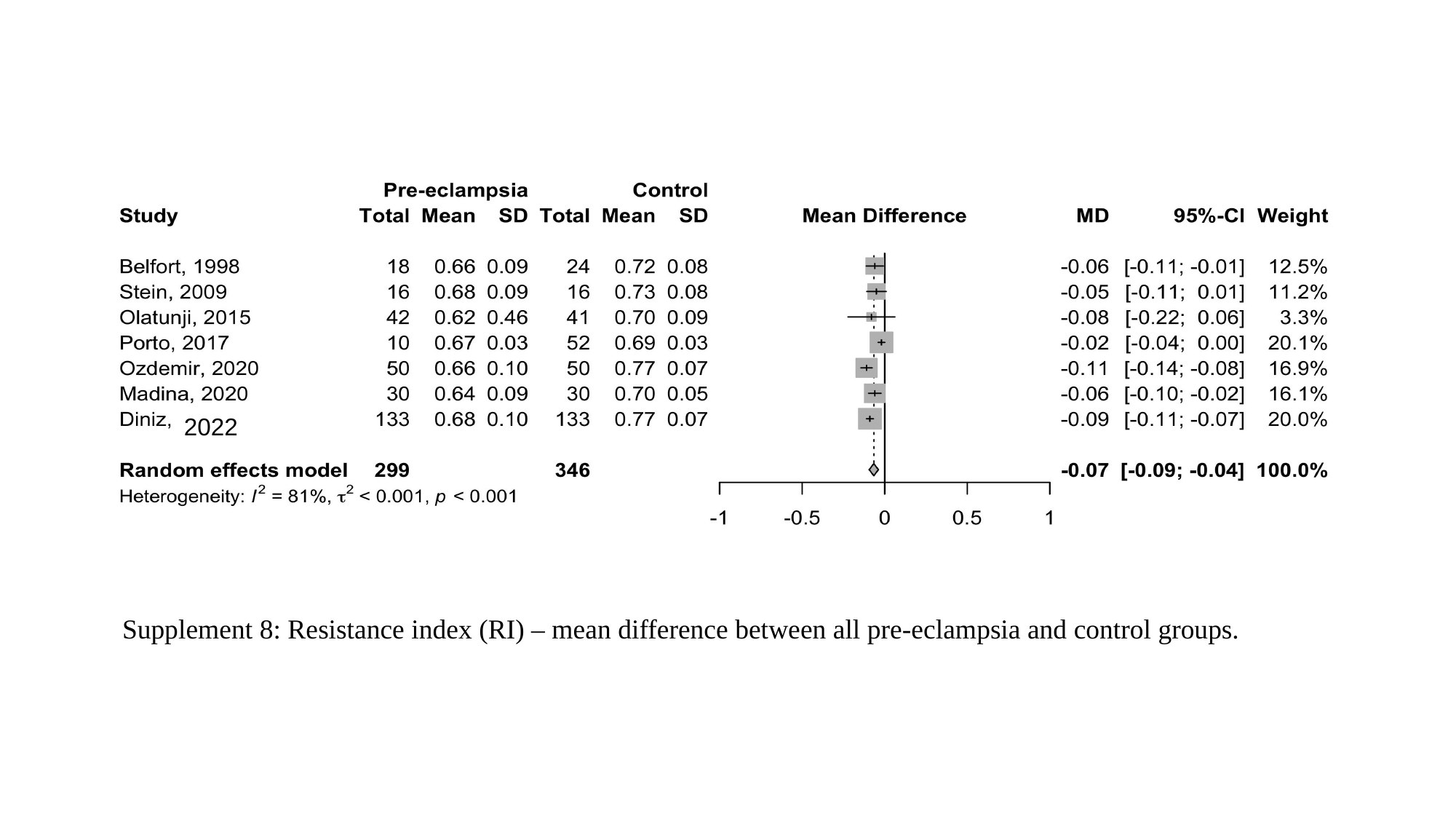

2022
Supplement 8: Resistance index (RI) – mean difference between all pre-eclampsia and control groups.
