# Supplementary material for: Ophthalmic artery Doppler in the complementary diagnosis of preeclampsia: a systematic review and meta-analysis
Source: BMC Pregnancy Childbirth. 2023 May 12;23:343. doi: 10.1186/s12884-023-05656-9 (PMC10176747; doi:10.1186/s12884-023-05656-9)
Supplement: Supplementary file 15 — Additional file 15. [file 12884_2023_5656_MOESM15_ESM.pptx]

## Slide 1
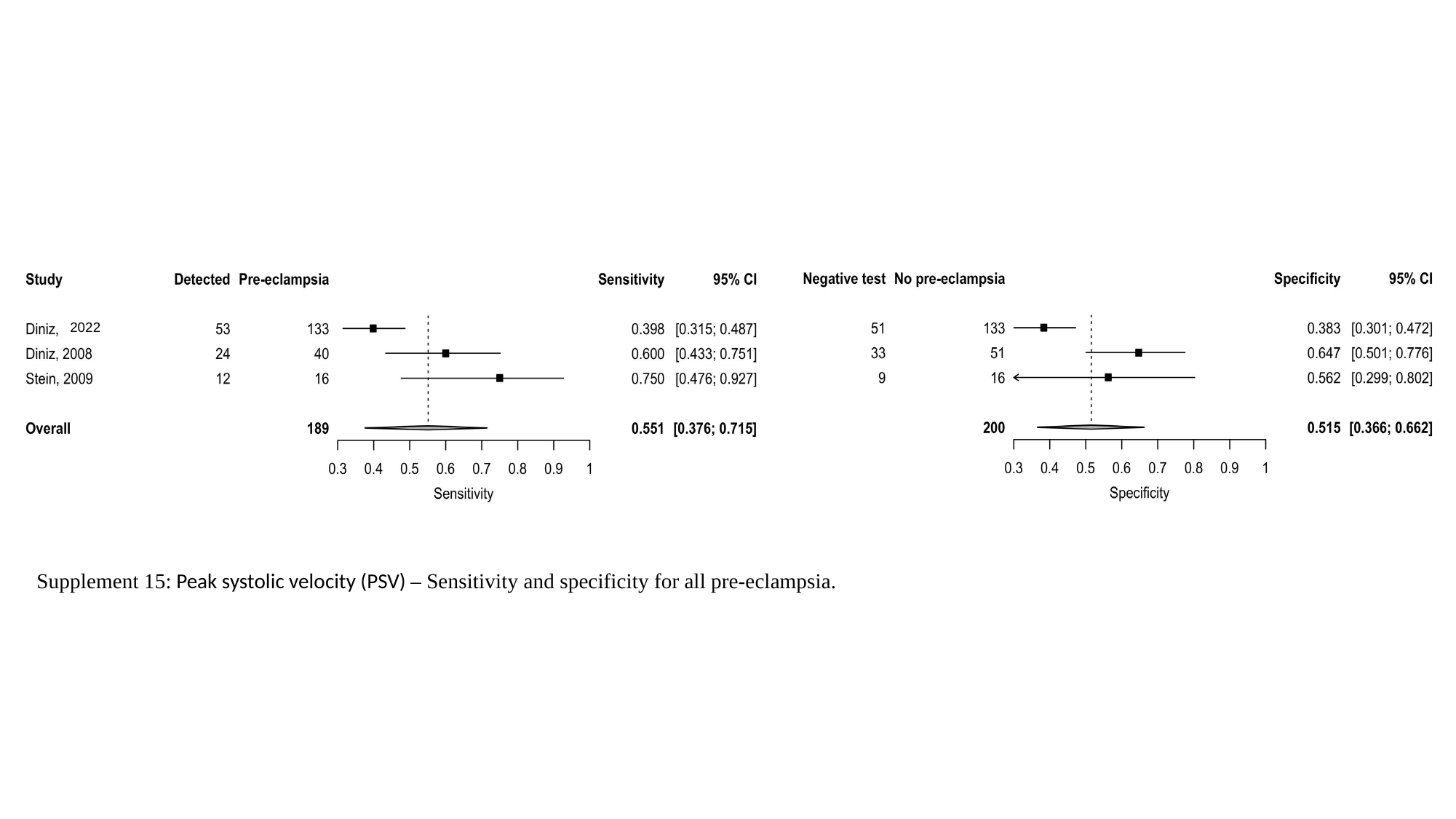

2022
Supplement 15: Peak systolic velocity (PSV) – Sensitivity and specificity for all pre-eclampsia.
