# Supplementary material for: Ophthalmic artery Doppler in the complementary diagnosis of preeclampsia: a systematic review and meta-analysis
Source: BMC Pregnancy Childbirth. 2023 May 12;23:343. doi: 10.1186/s12884-023-05656-9 (PMC10176747; doi:10.1186/s12884-023-05656-9)
Supplement: Supplementary file 16 — Additional file 16. [file 12884_2023_5656_MOESM16_ESM.pptx]

## Slide 1
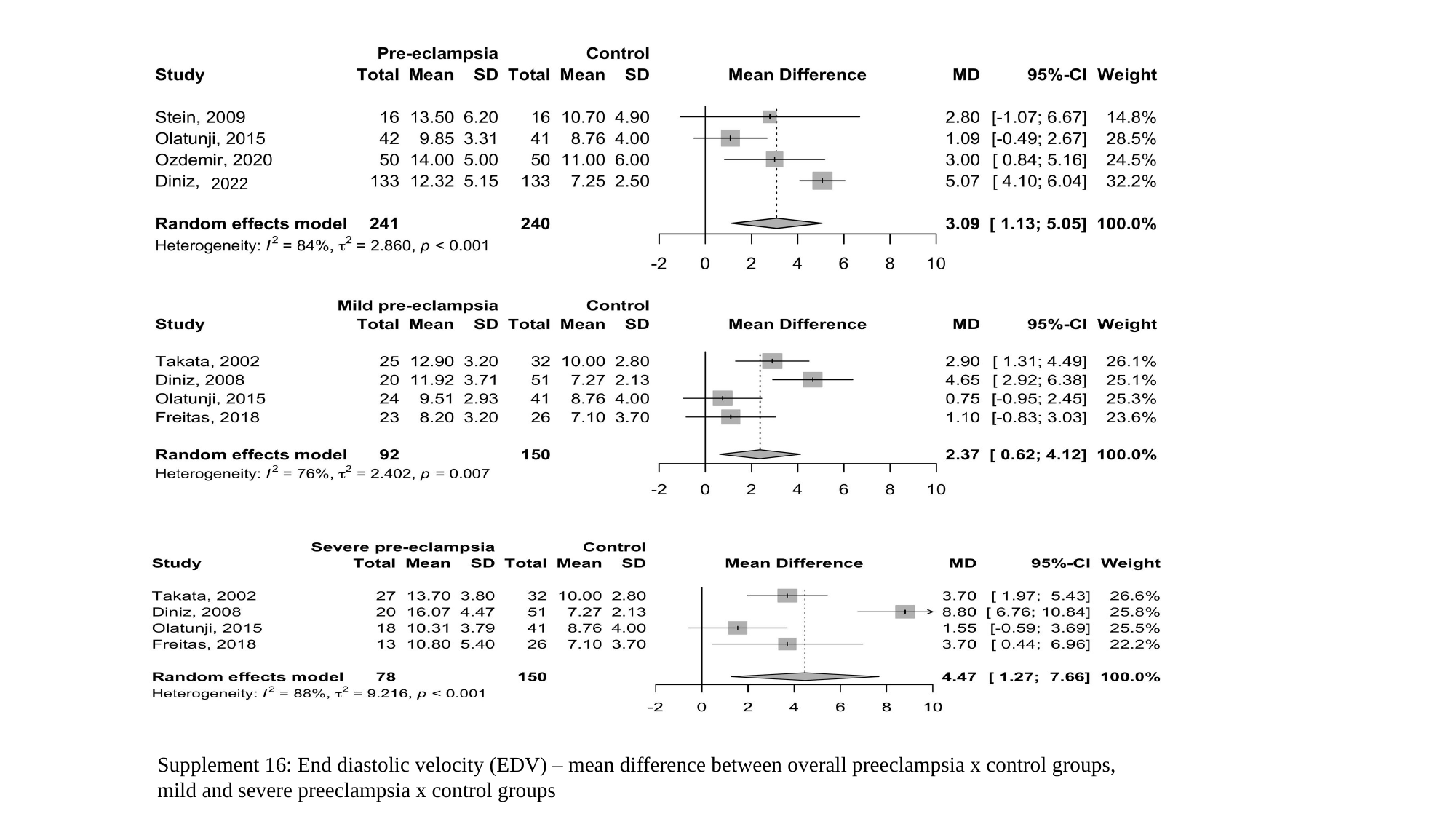

2022
Supplement 16: End diastolic velocity (EDV) – mean difference between overall preeclampsia x control groups, mild and severe preeclampsia x control groups
